# Affect-focused psychodynamic psychotherapy for mothers diagnosed with cancer – A feasibility study

**DOI:** 10.1016/j.invent.2026.100916

**Published:** 2026-02-16

**Authors:** Astrid Kuylenstierna, Maria Romare Strandh, Greta Melzi, Henrik Lindman, Ylva Hellstadius, Camilla Sköld, Lisa Ljungman, Anna Wikman

**Affiliations:** a Department of Women's and Children's Health, Uppsala University, Uppsala, Sweden; b Centre for Women's Mental Health during the Reproductive Lifespan (WOMHER), Uppsala University, Uppsala, Sweden; c Department of Immunology, Genetics and Pathology, Cancer Immunotherapy, Uppsala University, Uppsala, Sweden; d Centre for Cancer Rehabilitation, Stockholm, Sweden; e Department of Immunology, Genetics and Pathology, Cancer Precision Medicine, Uppsala University, Uppsala, Sweden

**Keywords:** neoplasms, parenting, psychological interventions, psychodynamic therapy, affect-focused psychotherapy

## Abstract

**Background:**

Parents with cancer face elevated psychological distress, often exacerbated by parenting responsibilities. Affect-Focused Psychodynamic Therapy (AFPT) has shown efficacy in improving emotion regulation, psychological well-being and self-compassion, but its feasibility and preliminary effect in this population remains unexplored.

**Objective:**

The aim of this study was to evaluate the feasibility, acceptability, safety, and preliminary effects on symptoms of depression and anxiety, of AFPT delivered via videoconferencing for parents with cancer.

**Methods:**

The intervention consisted of 10 sessions of AFPT, specifically affect phobia therapy. Qualitative data were collected through post-intervention interviews and analysed using inductive content analysis. Quantitative data were collected through self-report questionnaires at pre-intervention, post-intervention, and at 6-month follow-up measuring symptoms of depression and anxiety (primary outcome), parenting concerns, emotion regulation, self-efficacy, adaptive affective functioning, closeness in the family and self-rated health. Quantitative data were analysed using dependent-samples *t*-tests, with Cohen's *d* for effect sizes, and McNemar tests.

**Results:**

Fifteen mothers with cancer participated in the study. Results demonstrated efficient recruitment, acceptable study procedures, complete retention, and a relevant and beneficial intervention rated 8.4/10 in helpfulness. Moreover, findings showed significant reductions in symptoms of depression (Cohen's *d* = 1.29) and of anxiety (Cohen's *d* = 1.06) from pre- to post-intervention, maintained at 6-month follow-up, together with improvements in a majority of the secondary outcomes.

**Conclusions:**

Videoconferencing AFPT appears feasible, acceptable, and safe to use for mothers with cancer, with promising preliminary effects in reducing psychological distress. These findings support further evaluation of the intervention to determine its efficacy in this population using a randomized controlled trial.

## Highlights

• Online-delivered AFPT is feasible, acceptable, and safe for mothers with cancer.
• Results show promising preliminary effects in reducing psychological distress.
• This feasibility study supports a randomized controlled trial evaluation.

## 1 Background

Cancer poses an increasing global health challenge, with nearly 20 million new cases diagnosed annually (Bray et al., 2024), including a growing incidence of early-onset cancer, defined as diagnosis before the age of 50 (Ugai et al., 2022). Globally, it is estimated that up to 24% of adults diagnosed with cancer are also parents of dependent children (Inhestern et al., 2021), highlighting the intersection of cancer and caregiving responsibilities as a critical area for clinical attention and support.

Parenting can serve as an additional and significant stressor for individuals affected by cancer, and has been described as one of the most challenging aspects of the cancer experience (Lundquist et al., 2020). Mothers, in particular, report a range of parenting-related concerns, including fears about their ability to fulfil their maternal role and adequately care for their children (Spiropoulos et al., 2023), which can elicit strong feelings of guilt (Tavares et al., 2022; Zhu et al., 2022). Other concerns include how to communicate with children about the illness (Loggers et al., 2019; Park et al., 2021), especially in the context of advanced cancer (Caparso et al., 2021), as well as worries about children's emotional coping and fears related to potential parental loss (Caparso et al., 2021; Matuszczak-Świgoń and Bakiera, 2021; Pholsena et al., 2024; Romare Strandh et al., 2025). Such parenting concerns are also associated with elevated levels of depression, anxiety, and stress (Johannsen et al., 2022; Kuswanto et al., 2024; Romare Strandh et al., 2024; Shin et al., 2023), as well as reduced parenting self-efficacy (Cessna et al., 2016; Moore et al., 2015), which can affect the well-being of the entire family system (Shin et al., 2023; Whisenant et al., 2023).

Despite the significant psychological burden faced by parents with cancer, their psychological support needs often remain unmet (Romare Strandh et al., 2025; Liénard et al., 2022; Newman et al., 2023). Although a limited number of interventions have been developed for this population (Liénard et al., 2022; Chong et al., 2024; Inhestern et al., 2016; Romare Strandh et al., 2023; Yeoh et al., 2024), these typically focus on psychosocial support through psychoeducation and parenting skills training (e.g. Eklund et al., 2022; Lewis et al., 2015; Palacios et al., 2023; Park et al., 2022; Phillips et al., 2022; Stafford et al., 2021). Psychotherapy has been identified as one of the few non-pharmacological treatments with demonstrated long-term efficacy in reducing depressive symptoms among adults with cancer (Coutiño-Escamilla et al., 2019). Numerous randomized controlled trials (RCTs) have studied the effects of Cognitive Behavioural Therapy (CBT) on symptoms of anxiety and depression among cancer survivors, with significant improvements during intervention and up to 6-month follow-up (Zhang et al., 2022). Psychodynamic psychotherapy (PDT), however, is much less studied within this population, despite its established efficacy in treating depression and anxiety in other clinical groups (Leichsenring et al., 2023; Leichsenring and Klein, 2014), with comparable effects to CBT and benefits that outweigh potential risks (Leichsenring et al., 2023; Lilliengren, 2023; Steinert et al., 2017).

Affect-Focused Psychodynamic Therapy (AFPT) is a modern, integrative form of therapy that blends psychodynamic theory with emotion-centred, experiential techniques and aspects of CBT. AFPT prioritises the activation, exploration, and resolution of emotional experiences (affects), particularly those that have been avoided, suppressed, or defended against, in order to reduce psychological symptoms and enhance emotional well-being (Diener and Hilsenroth, 2009; McCullough et al., 2003; Svebeck, 2024). In the context of cancer—characterised by significant emotional burden and potentially diminished psychological resilience—therapeutic approaches that directly target emotional processing may be particularly beneficial. Importantly, emotion regulation plays a central role in the development and maintenance of psychiatric disorders (McCullough et al., 2003). Previous research has shown that emotion regulation strategies such as emotional suppression are associated with increased psychological distress among cancer survivors (Baziliansky and Cohen, 2021), underscoring the relevance of targeting these processes in therapeutic work.

AFPT has demonstrated long term efficacy in clinical populations (Lilliengren et al., 2025), but has not yet been evaluated among cancer patients. Remote face-to-face therapy via videoconferencing may increase accessibility for individuals experiencing cancer-related fatigue, logistical barriers, or geographical distance from treatment providers, making an evaluation of videoconferencing AFPT in this population warranted. Previous studies indicate that AFPT for clinical populations can be effectively delivered online using a guided self-help format (Johansson et al., 2017; Lindqvist et al., 2020; Mechler et al., 2024; Mechler et al., 2022), suggesting that remote delivery is feasible. Online AFPT has shown symptom reduction of depression and anxiety (Johansson et al., 2017; Lindqvist et al., 2020; Mechler et al., 2024; Mechler et al., 2022) as well as improved emotion regulation and self-compassion (Lindqvist et al., 2020), two factors that are positively associated with psychological well-being among parents with cancer (Babore et al., 2019; Peh et al., 2017).

Given the high psychological burden among parents with cancer and the potential of AFPT to improve emotion regulation and mental health, evaluating AFPT in this population is of significant clinical interest. This study therefore aimed to assess the feasibility, acceptability, safety, and preliminary effects on symptoms of depression and anxiety of AFPT via videoconferencing for parents with cancer.

## 2 Methods

### 2.1 Participants

Participants were recruited via advertisements on a project website and through social media platforms (Facebook and Instagram) between May and September 2024. Inclusion criteria were: (1) a cancer diagnosis within the past five years, (2) being a parent of at least one child aged 18 or younger at the time of diagnosis, and (3) experiencing psychological distress related to parenting and cancer. Exclusion criteria included: (1) serious psychiatric conditions such as severe depression or suicidal ideation, assessed through clinical evaluation using the Montgomery-Åsberg Depression Rating Scale (MADRS-S) (Montgomery and Åsberg, 1979), and/or (2) ongoing psychotherapy. Interested individuals completed an online yes/no eligibility screening form on the project website and provided their contact details. Eligible participants were then contacted, sent detailed study information, and invited to a full eligibility assessment. These assessments were conducted via videoconference by a licensed psychologist (GM), during which participants received verbal study information, were interviewed regarding inclusion and exclusion criteria, completed the MADRS-S, and reported their psychiatric history and any potential comorbidities. Informed consent was obtained online using REDCap (Harris et al., 2009; Harris et al., 2019), a secure web-based application hosted by Uppsala University. The study was approved by the Swedish Ethical Review Authority (reference number: 2024-01399-01).

### 2.2 Intervention and data collection

The intervention consisted of 10 sessions of AFPT, specifically affect phobia therapy (McCullough et al., 2003), each lasting 50 min. Participants were assigned to one of four licensed psychotherapists with a post graduate diploma in psychotherapy with a therapeutic focus on APT, who delivered the sessions via videoconferencing. The therapists conducted individual case conceptualization for each participant, instead of strictly focusing on cancer and parenting. Upon completion of the intervention, participants took part in individual videoconference interviews exploring their experiences of the study and the intervention. These interviews were conducted by a licensed psychologist (GM) using a semi-structured interview guide (Supplementary Material). Interviews lasted an average of 34 min (range: 16–47 min), were audio-recorded, and transcribed verbatim by a professional transcriber.

To evaluate preliminary effects of the intervention, quantitative assessments were conducted using REDCap at three time points: pre- and post-intervention, and at 6-month follow-up. At pre-intervention, participants also completed demographic questions, and at post-intervention, they rated their overall satisfaction with the intervention in terms of how well it met their needs, using a scale from 0 to 10 (0 = not helpful at all; 10 = very helpful).

### 2.3 Outcomes

Both qualitative and quantitative data were collected to evaluate the feasibility, acceptability, safety, and preliminary effect of the intervention.

#### 2.3.1 Feasibility, acceptability and safety

*Feasibility* was assessed by examining barriers and facilitators related to study procedures and intervention completion. This included the ability to recruit a sufficient number of participants, participant adherence to the intervention, and the feasibility of delivering the intervention and collecting pre-, post-, and follow-up data as planned. *Acceptability* was explored through participants' perceptions of the intervention. Specific areas of interest included whether the intervention was perceived as helpful, understandable, and relevant to the target population, as well as whether participants would recommend it to others. Additional aspects assessed included overall satisfaction, perceived benefits, and the intervention's impact on participants' mental health. *Safety* was monitored by therapists observing the presence of any adverse effects potentially associated with the intervention such as serious psychiatric symptoms, such as major depression or suicidal ideation. While some emotional distress was anticipated as a normal part of the therapeutic process, participants were specifically asked during interviews whether they had experienced any negative effects attributed to the intervention.

#### 2.3.2 Preliminary effect of the intervention

##### 2.3.2.1 Primary outcomes

Primary outcomes were symptoms of depression and anxiety measured using the Patient Health Questionnaire (PHQ-9) (Kroenke et al., 2001) and the Generalised Anxiety Disorder Questionnaire (GAD-7) (Spitzer et al., 2006), respectively. PHQ-9 consists of nine items on a 4-point Likert scale from 0 (*not at all*) to 3 (*nearly every day*), with a total score of between 0 and 27. Scores of 10 and above indicate moderate-severe symptoms and were considered a clinical cut-off for depression in this study. PHQ has previously demonstrated construct validity, test-retest reliability and excellent internal consistency (α = 0.86–0.89) (Kroenke et al., 2001), in the present study, it showed acceptable internal consistency (α = 0.71). GAD-7 includes seven items on a 4-point Likert scale from 0 (*not at all*) to 3 (*nearly every day*), with total scores ranging from 0 to 21. Scores of 10 and above represent moderate-severe symptoms and were considered a clinical cut-off for anxiety in this study. GAD-7 has previously shown construct validity, good test-retest reliability (intraclass correlation = 0.83) and excellent internal consistency (α = 0.92) (Spitzer et al., 2006), with an acceptable internal consistency in this study (α = 0.68).

##### 2.3.2.2 Secondary outcomes

*Parenting concerns:* The Parenting Concerns Questionnaire (PCQ) (Ljungman et al., 2024; Muriel et al., 2012) was used to identify parenting-related worries in parents diagnosed with cancer. It includes 15 items assessing concerns about practical impact of illness on the child/ren, emotional impact of illness on the child/ren, and concerns about the co-parent, measured on a 5-point Likert scale ranging from 1 *(not at all concerned)* to 5 *(extremely concerned)*. The average of the total scores can range from 1 to 5, with higher scores indicating greater parenting concerns. PCQ has previously demonstrated convergent validity and excellent internal consistency (α = 0.90) (Ljungman et al., 2024), with good internal consistency in this study (α = 0.81).

*Emotion regulation:* Emotion regulation was measured using the Emotion Regulation Questionnaire (ERQ) (Preece et al., 2020), which consists of 10 items assessing differences in two strategies: *cognitive reappraisal* and *expressive suppression*. Cognitive reappraisal involves changing one's interpretation of a situation in order to alter the emotional response, such as viewing a stressful experience as an opportunity for growth. In contrast, expressive suppression involves inhibiting or reducing the outward expression of emotions. Participants rated their responses on a 7-point Likert scale, ranging from 1 (*strongly disagree*) to 7 (*strongly agree*). Total scores range from 6 to 42 for cognitive reappraisal (6 items) and 4 to 28 for expressive suppression (4 items), with higher scores indicating a greater tendency to use the specific strategy. ERQ has previously shown convergent validity and acceptable to excellent levels of internal consistency (cognitive reappraisal α = 0.89–0.90; expressive suppression α = 0.76–0.80) (Preece et al., 2020). In this study the internal consistency was good (cognitive reappraisal α = 0.89; and expressive suppression α = 0.82).

*Self-efficacy*: Self-efficacy was assessed using the General Self-Efficacy Scale (GSE) (Schwarzer and Jerusalem, 2012), which includes 10 items that evaluate an individual's confidence in their ability to handle difficult situations. Participants rated their responses on a 4-point Likert scale, ranging from 1 (*not true at all*) to 4 (*exactly true*). The total score ranges from 10 to 40, with higher scores reflecting greater self-efficacy. GSE has previously demonstrated concurrent validity and acceptable to excellent levels of internal consistency (α = 0.75 to 0.90) (Schwarzer and Jerusalem, 2012), with a good level of internal consistency in this study (α = 0.83).

*Affect Phobia Test*: The ability to experience, express and manage emotions, and use adaptive affective functioning was assessed with the Affect Phobia Test (Frankl et al., 2016), which consists of 20 items on a 5-point Likert scale ranging from 1 (*not at all*) to 5 (*very much*). Total scores range from 20 to 100, with a higher score indicating better adaptive affective functioning. The Affect Phobia Test has previously demonstrated convergent validity and good internal consistency (α = 0.88) (Frankl et al., 2016), similar to the internal consistency in this study (α = 0.86).

*Closeness in the family*: To assess closeness in the family, an adapted version of the Adult Child Relationship Scale (ACRS) (Criss and Shaw, 2005) was used. Participants responded to 5 items on a 5-point Likert scale from 1 (*definitively not*) to 5 (*definitively*) with a total score ranging from 5 to 25. Higher scores indicate more closeness and warmth in the relationship with the child. Closeness in the family has demonstrated good levels of internal consistency both previously (α = 0.85) (Wetterborg et al., 2019) and in this study (α = 0.86).

*Self-rated health*: Self-rated health (SRH) was assessed using a single item: “How is your health in general?”. Participants responded on a 5-point Likert scale with the alternatives 1 (*very good)*, 2 *(good)*, 3 (*fair)*, *4 (bad)*, 5 (*very bad) (*Robine and Jagger, 2003*).* For the presentation of participant characteristics in Table 1, SRH was dichotomized into good (*very good, good*) and poor (*fair, bad, very bad*).

**Table 1:** Participant characteristics (*n* = 15).

Participant characteristics	n (%) or mean [SD]
Age, years (range)	45 (31–54) [4.88]
Parent	
Mother	15 (100)
Education level	
Primary education	1 (7)
Post-secondary education	14 (93)
Occupation^a^	
Working full-time	4 (27)
Working part-time	5 (33)
On sick leave	9 (60)
Unemployed	1 (7)
Civil status	
Single	2 (13)
In a partnered relationship	13 (87)
Relationship length	
2–10 years	3 (23)
> 10 years	10 (77)
Children	
No. of children ≤18 years per participant	2.07 [0.59]
Age youngest child	7.20 [3.48]
Cancer type^b^	
Breast cancer	10 (67)
Ocular melanoma	1 (7)
Ovarian cancer	1 (7)
Leiomyosarcoma	1 (7)
Colorectal cancer	1 (7)
Adrenal cortex cancer	1 (7)
Time since diagnosis, years	1.27 [1.62]
Self-reported cancer status	
Treatment status	
Under treatment	11 (73)
Completed treatment	4 (27)
Curable	
Yes	13 (87)
No	2 (13)
Cancer recurrence (yes)	3 (20)
Self-rated health	
Good	1 (7)
Poor	14 (93)
Self-reported mental health status	
Psychological distress before cancer diagnosis	
Yes	11 (73)
No	4 (27)
Self-reported psychiatric diagnosis before cancer, yes	3 (20)
Diagnosis^a^	
Depression	2 (13)
PTSD	1 (7)
Anxiety	1 (7)
Social anxiety	1 (7)
Panic disorder	1 (7)
Burnout	1 (7)
Psychological distress after cancer diagnosis, yes	15 (100)
New psychiatric diagnosis after cancer diagnosis, yes	1 (7)
Diagnosis^a^	
Burnout	1 (7)

a Participants could fill in multiple responses.

b Numbers do not add up to 100% due to the rounding of decimals.

### 2.4 Data analysis

Qualitative data were analysed by two of the authors (AK and MRS) using inductive qualitative content analysis, following the methodology described by Graneheim and Lundman (2004). Although the semi-structured interview guide focused on predefined areas of feasibility, acceptability and safety, an inductive approach was chosen for the analysis to allow for more open interpretations of the data. This approach enabled the identification of unexpected issues, barriers, facilitators, or participant needs that could influence feasibility, acceptability and safety. The analysis began with repeated readings of the transcribed interviews to gain a comprehensive understanding of the content. Meaning units, phrases or sentences relevant to the research questions, were identified, condensed while preserving core meaning, and assigned codes representing key concepts. Codes with similar content were then grouped into sub-categories, which were further organized into broader categories. The analysis process was iterative, with ongoing discussions and reviews to ensure that the categories accurately reflected the data.

Descriptive statistics were used to present participant characteristics and study procedures. To assess changes in outcomes from pre- to post-intervention, and from post-intervention to follow-up, dependent-samples *t*-tests were conducted. Effect sizes of within-group changes between pre- and post-intervention were calculated using Cohen's d. The McNemar test was employed to analyse changes in binary outcomes (i.e., scoring above or below the clinical cut-off for depression and anxiety) between pre, post and follow-up. Given the absence of a control group, the small sample size, and the use of unadjusted analyses, the quantitative findings should be regarded as exploratory and hypothesis-generating rather than confirmatory. These analyses were intended to complement the qualitative data on feasibility, acceptability and safety and provide preliminary insights into potential intervention effects. All statistical analyses were performed using IBM SPSS Statistics, Version 28.

## 3 Results

### 3.1 Participant characteristics

**Fig. 1:**
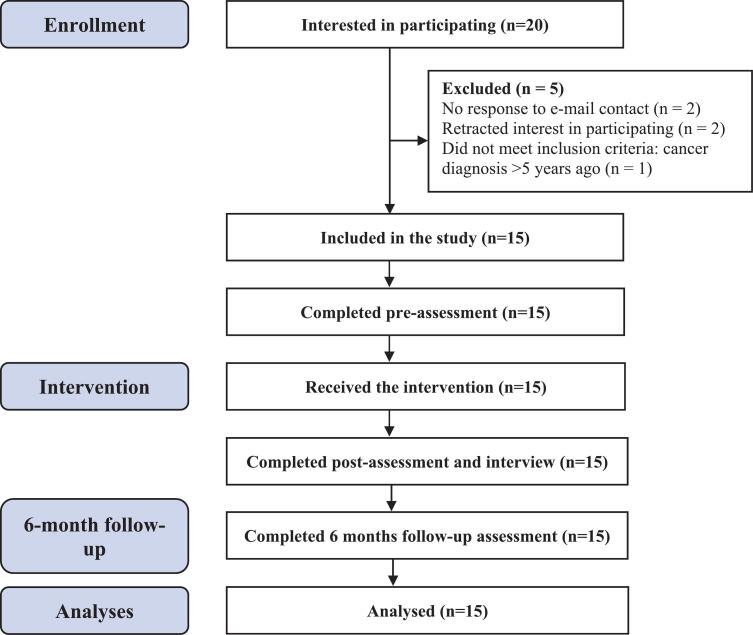
Flowchart of the study process.

Although recruitment targeted parents with cancer, mainly mothers with cancer expressed an interest in participating (mothers *n* = 19, fathers n = 1). Of these, 16 mothers underwent eligibility assessment, and 15 met the inclusion criteria and were enrolled in the study (Fig. 1). All participants completed pre-, post- and 6-month follow-up assessments. One participant chose to discontinue the intervention after eight sessions, reporting sufficient benefit.

The characteristics of study participants are shown in Table 1. All were mothers aged between 31 and 54 years old (mean 45 [*SD* = 4.88]), and the majority (87%) were in a partnered relationship at pre-intervention assessment. Two-thirds (67%) had been diagnosed with breast cancer, but several other cancer diagnoses were represented. The mean number of dependent children (≤18 years) per participant was 2.07 (*SD* = 0.59) and the age of the youngest child was 7.2 years (*SD* = 3.48).

### 3.2 Feasibility, acceptability and safety – qualitative findings

The content analysis resulted in three categories and seven sub-categories describing participants' experiences of the study and the intervention. See Table 2 for an overview and illustrative quotes.

**Table 2:** Overview of categories and subcategories with examples of quotes.

Categories	Subcategories	Quotes
Study design and intervention delivery	Navigating study participation	*“I think this fast process was really good, /…/ you constantly felt taken care of and informed, /…/ it just went smoothly.” -* Mother #1
	Reflections on intervention format	*“Ten sessions sounded like a lot at first, but by the end, we realized, “Wow, that went by so fast, and there's still so much to talk about. So from the patient's perspective, you just want to keep going [laughs], because… well, the problems continue, and everything related to the illness goes on.”* - Mother #12
Perceptions of intervention content	Intervention triggered emotional experiences	*“I don't think I've ever cried as much as I have during these weeks of therapy, both during the sessions and in between. But it has been really relieving. It has been tough, but it has been positive”* – Mother #10
	Focus of therapy sessions	*“I thought it was good that the focus was on many parts. It's not just one specific thing, like I have anxiety because I have cancer, but also the parental aspect and… yes, everything. You don't just have one problem [laughs]; there are many parts that can get tangled together. I felt that I could bring up all aspects, which was good.”–* Mother #12
Perceived effects of the intervention	Impact on psychological well-being	*“This psychotherapy has taken me to a place I have never been in my life before. It has been absolutely amazing [laughs]. /…/ I would say that it has made me feel really good and allowed me to feel very confident in myself.”* - Mother #14
	Acquiring new emotion regulation strategies	*“But I think I've somewhat understood how my childhood has affected me as a parent, and how the illness has affected me as a parent. Because we've come back to the illness, even though we've talked about my childhood and things that happened there, and my parents' parenting and so on.”* Mother #19
	Strengthening family relationships	*“The children have noticed that I have been more relaxed and happier, and therefore they have turned to me more /…/ It was a powerful effect, I did not expect the therapy to have an impact on my relationship with the children.” -* Mother #9

#### 3.2.1 Study design and intervention delivery

##### 3.2.1.1 Navigating study participation

Participants found the inclusion process easy and straightforward, the study information adequate and expressed feeling well supported throughout, though some wanted more detailed information about AFPT as a method and its emotional demands. The timing of the intervention in relation to diagnosis and treatment shaped participants experiences of participation. While those in active treatment used therapy to manage acute distress related to e.g., hospital visits and fear of death, those in post-treatment described psychological support needs as more prominent than earlier on, and at the same time feeling more emotionally available to engage in therapy. Questionnaires were viewed as relevant and manageable, though a few participants found some items repetitive or unclear, and noted that their mood and stage of cancer treatment influenced responses. Post-intervention interviews were appreciated for allowing deeper reflection beyond the questionnaires, despite some difficulty recalling early study experiences due to the time gap between enrolment and the interview.

##### 3.2.1.2 Reflections on intervention format

Participants generally appreciated the weekly session format, finding the pacing supportive of reflection, continuity and engagement with the therapeutic process. Regarding the duration of the intervention there were mixed experiences. While some found the ten sessions sufficient to build a therapeutic relationship and understand the method, others found it too short to address deeper issues or to manage emotions stirred up in therapy. One participant reported feeling satisfied after eight sessions and opted to discontinue early. Some experienced the ending as abrupt and suggested adding a follow-up session. A few wished to continue therapy with the same therapist but were unable to continue due to the design of the study. The videoconferencing format was regarded as convenient, accessible, conducive to privacy, and especially helpful for those experiencing fatigue or logistical challenges. Being at home enhanced comfort and a sense of safety for many. Challenges included the need for a private space, occasional technical issues, and, for some, difficulty forming an emotional connection via a screen.

#### 3.2.2 Perceptions of intervention content

##### 3.2.2.1 Intervention triggered emotional experiences

Participants found the intervention meaningful, valuing the experience of exploring their thoughts, emotions and behaviours in the context of parenting and cancer. Support in facing difficult feelings was appreciated, though the therapeutic work was often emotionally challenging. Several participants likened the experience to a “rollercoaster,” noting that the sessions could evoke intense, unexpected emotions, leaving some feeling drained, an effect heightened by cancer-related fatigue or cognitive challenges. Despite discomfort in confronting painful emotions and shifting maladaptive patterns, most participants viewed these difficulties as temporary and manageable, with benefits outweighing the strain. One participant highlighted that the therapy method demands emotional readiness and willingness to face challenging feelings.

##### 3.2.2.2 Focus of therapy sessions

Participants valued the intervention's holistic focus on parenting in the context of cancer, noting benefits for emotion regulation, coping, and an improved ability to support their children. While the client-led session format was appreciated for its flexibility, some participants felt parenting was not fully addressed and desired more therapist guidance in staying focused on the topic. Some also experienced uncertainty about the overall therapeutic goals.

#### 3.2.3 Perceived effects

##### 3.2.3.1 Impact on psychological well-being

Participants generally reported improved psychological well-being during and after the intervention, including feeling calmer, less irritable, and experiencing a general sense of emotional stability, with one participant noting they were “in a better place now than before.” Most experienced no adverse effects, though one participant described a decline in psychological well-being, attributing it to heightened emotional awareness but feeling uncertain whether it stemmed from the intervention or the progression of the cancer.

##### 3.2.3.2 Acquiring new emotion regulation strategies

Participants reported gaining deeper understanding and acceptance of their thoughts, emotions, and behaviours, learning to contextualize emotional responses and develop self-compassion, particularly in the emotionally demanding context of cancer. They acquired practical skills for facing, rather than supressing, difficult emotions, regulating emotions, improving emotional communication, and reducing unhelpful rumination by consciously shifting thought processes. Many described increased confidence, resilience, and assertiveness, including prioritizing their needs and managing worry more effectively. All participants expressed they would recommend the intervention to other parents with cancer, citing its benefits for dealing with their emotions, parenting support, self-compassion, and reducing loneliness. A strong therapeutic alliance was highlighted as a key factor enhancing trust, openness, and the intervention's effectiveness.

##### 3.2.3.3 Strengthening family relationships

Participants described the intervention as beneficial not only for their individual well-being but also in fostering positive changes within their family dynamics. Several reported improvements in their relationships with their children, including being more patient, emotionally present, and open in expressing their own feelings. These changes were perceived to have a ripple effect, with children becoming more comfortable discussing their emotions, appearing less worried, expressing greater happiness and contentment, and showing increased interest in spending time with their mothers. Positive changes were also noted in couple relationships. Participants described enhanced emotional understanding of their partners and improvements in communication, which contributed to a stronger sense of connection within the relationship.

### 3.3 Feasibility, acceptability, safety, and preliminary effect – quantitative findings

One key feasibility indicator was the efficiency of the recruitment process. Due to strong interest in the study, all 15 participants were successfully enrolled within 25 days of advertising. All participants completed the pre-, post- and 6-month follow-up assessments, with a mean questionnaire completion time of 16 min (range: 5–58 min; median: 14) across all timepoints. In terms of acceptability, participants were asked to rate their overall satisfaction with the extent to which the intervention met their needs. Fourteen participants provided ratings on a scale from 0 to 10, with scores ranging from 6 to 10, and a mean score of 8.4, indicating a high level of overall satisfaction. One participant did not respond to this question.

#### 3.3.1 Preliminary effects and safety

##### 3.3.1.1 Primary outcomes – symptoms of depression and anxiety

Depressive symptoms significantly decreased from pre- to post-intervention t(14) = 5.01, *p* < 0.001, Cohen's *d* = 1.29, with no change from post- to 6-month follow-up assessment t(14) = −0.617, *p* = 0.547. Similarly, for anxiety, there was a significant reduction in scores at post-intervention, t(14) = 4.11, *p* = 0.001, Cohen's *d* = 1.06, which was maintained at 6-month follow-up, t(14) = −0.221, *p* = 0.828. This suggests that the intervention is preliminarily effective in decreasing symptoms of depression and anxiety among mothers diagnosed with cancer, also indicating a safe intervention with no adverse effects. See Table 3 for a summary of results.

**Table 3:** Changes in primary and secondary outcomes across timepoints (n = 15).

		Change from previous time point^a^		
Measurement		Mean [SD]	*p*-value	Cohen's *d*^b^
Depression (PHQ9)	Pre	11.93 [4.32]		
	Post	6.20 [2.80]	<0.001⁎⁎	1.29
	Follow-up	7.07 [5.91]	0.547	−0.16
Anxiety (GAD7)	Pre	9.47 [3.34]		
	Post	5.73 [3.37]	0.001⁎	1.06
	Follow-up	5.93 [2.89]	0.828	−0.06
Parenting concerns (PCQ)	Pre	2.84 [0.59]		
	Post	2.08 [0.59]	<0.001⁎⁎	1.21
	Follow-up	2.22 [0.66]	0.379	−0.23
Cognitive reappraisal (ERQ)	Pre	25.13 [7.35]		
	Post	28.60 [4.90]	0.034⁎	−0.61
	Follow-up	26.20 [5.83]	0.117	0.43
Expressive suppression (ERQ)	Pre	13.67 [5.01]		
	Post	11.53 [4.84]	0.247	0.31
	Follow-up	12.87 [4.21]	0.406	−0.22
General self-efficacy (GSE)	Pre	26.13 [4.05]		
	Post	28.73 [3.45]	0.028⁎	−0.63
	Follow-up	27.47 [4.44]	0.131	0.41
Affect Phobia Test	Pre	66.47 [10.20]		
	Post	71.07 [10.26]	0.042⁎	−0.58
	Follow-up	64.87 [12.70]	0.005⁎	0.85
Closeness in the family^c^	Pre	18.36 [4.62]		
	Post	20.93 [2.34]	0.059	−0.55
	Follow-up	20.73 [3.28]	0.755	0.08
Self-rated health (SRH)	Pre	3.20 [0.56]		
	Post	2.67 [0.72]	0.006⁎	0.83
	Follow-up	2.53 [0.74]	0.499	0.18

a Post- compared to pre-intervention, and 6-month follow-up compared to post-intervention.

b Cohen's *d* effect size = 0.20 small, 0.50 medium, and 0.80 large.

c *n* = 14, one participant had no children >5 years and did not fill in the questionnaire.

⁎ *p* < 0.05.

⁎⁎ *p* < 0.001.

McNemar's tests were conducted to examine changes in the proportions of participants scoring above and below the clinical cut-off for depression and anxiety pre- and post-, and post- to 6-month follow-up (Table 4). Results indicated a significant decrease in the proportion of participants scoring above the cut-off on depression (*n* = 10, 66% and *n* = 2, 13%, respectively) and anxiety (*n* = 8, 53% and n = 2, 13%, respectively) from pre- to post-intervention, with proportions remaining stable at 6-month follow-up.

**Table 4:** Clinically significant changes in primary outcomes across time points.

			Change from previous time point^a^
Measurement	n(%) > cut-off		*p*-value^c^
Depression (PHQ9)^b^	Pre	10 (66)	
	Post	2 (13)	0.008⁎
	Follow-up	5 (33)	0.250
Anxiety (GAD7)^b^	Pre	8 (53)	
	Post	2 (13)	0.031⁎
	Follow-up	1 (7)	1.000

a Post- compared to pre-intervention, and 6-month follow-up compared to post-intervention.

b Cut-offs were set at ≥10 for depressive symptoms and ≥ 10 for anxiety.

c Significance in difference in no. of participants was computed using McNemar test.

⁎ *p* < 0.05.

##### 3.3.1.2 Secondary outcomes

Significant improvements were observed across all but two of the secondary outcomes following the intervention. These included reductions in parenting concerns (t(14) = 4.70, *p* < 0.001, Cohen's *d* = 1.21) and affect phobia (t(14) = −2.24, *p* = 0.042, Cohen's *d* = −0.58), as well as increases in cognitive reappraisal (t(14) = −2.35, *p* = 0.034, Cohen's *d* = −0.61), self-efficacy (t(14) = −2.45 *p* = 0.028, Cohen's *d* = −0.63), and self-rated health (t(14) = 3.23, *p* = 0.006, Cohen's *d* = 0.83). No significant changes in expressive suppression (t(14) = 1.21, *p* = 0.247, Cohen's *d* = 0.31) and closeness in the family (t(13) = −2.07, *p* = 0.059, Cohen's *d* = −0.55) were found post-intervention. At 6-month follow-up, improvements in outcomes remained stable with exception for scores on the affect phobia test, which decreased from post-intervention (t(14) = 3.297, *p* = 0.005). These findings suggest that the intervention had a broad positive preliminary impact, improving various psychological and relational domains for participants both in the short- and longer-term.

## 4 Discussion

This study yields promising findings: videoconferencing AFPT appears feasible, acceptable, and safe for mothers with cancer, with preliminary effects in reducing psychological distress. The results demonstrate efficient recruitment, acceptable study procedure, complete retention, and an overall satisfaction with the intervention. The intervention was perceived as relevant and beneficial, such as offering valuable support in facing difficult emotions and helping participants acquire new strategies. Participants also suggested areas for improvement, including a stronger emphasis on parenting issues, better preparation for emotional challenges, and the addition of a follow-up session. Moreover, the pre-, post- and 6-month follow-up assessments showed significant improvements in symptoms of anxiety and depression, as well as in most of the secondary outcomes, indicating a broad positive preliminary impact, improving various psychological and relational domains both in the short- and longer-term.

### 4.1 Feasibility markers

Facilitators related to study procedures and intervention completion included the efficient recruitment and inclusion process, data collection methods resulting in that all participants completed all assessments, and the videoconferencing format of delivery. One barrier was that some participants had difficulty interpreting certain questionnaire items, underscoring the need for improved clarity in further studies. All participants completed the intervention, and the online format likely supported adherence by reducing travel demands, which is an important advantage for individuals with cancer who often experience limited energy. At the same time, because videoconferencing more closely resembles face-to-face treatment than other online formats, it may help maintain elements of in-person therapy. These findings are consistent with previous studies of videoconferencing psychotherapy demonstrating feasibility (Nurtsch et al., 2025; Laczkovics et al., 2023), and further underscore the suitability of videoconferencing as a delivery format for psychodynamic interventions. One notable feasibility find was the inclusion of only mothers with cancer. Although recruitment targeted parents with cancer, interest was predominantly expressed by mothers. This lends to question whether this form of support appeals to fathers with cancer, or whether more intensive recruitment efforts must be made to ensure reaching all parents with cancer experiencing a need for support. A potential gender bias in social media engagement and advertisement algorithms may partly explain the imbalanced representation, underscoring the need for diverse recruitment strategies in future studies.

### 4.2 Acceptability markers

The intervention was generally perceived as acceptable, with participants reporting it as helpful, relevant, and beneficial for their mental health and family. They rated its usefulness highly (mean 8.4/10) and would recommend it to other parents with cancer. While some found it emotionally challenging, partly due to cancer-related fatigue, such short-term distress is consistent with prior AFPT studies (Lindqvist et al., 2020). When designing interventions for this population, it is essential to account for additional cancer-related stressors and to inform participants about potential emotional responses to minimize burden.

Participants reported improved psychological well-being during and after the intervention, including greater insight, emotion regulation, and self-compassion. These outcomes align with one previous study of AFPT (Lindqvist et al., 2020) and are particularly relevant given the heightened emotional burden faced by parents with cancer (Matuszczak-Świgoń and Bakiera, 2021; Romare Strandh et al., 2025). Participants also noted enhanced relationships with their children, suggesting potential benefits for the wider family, consistent with prior research (Shin et al., 2023; Whisenant et al., 2023).

The intervention was considered relevant, providing emotional tools and support, though some participants found its structure unclear and desired a stronger focus on parenting. Given that parenting is associated with psychological distress among parents with cancer (Johannsen et al., 2022; Kuswanto et al., 2024), the expressed desire underscores the unmet support needs documented in this population (Romare Strandh et al., 2025). However, individual needs guided the session content since the intervention was not manualised. This suggests that, for some parents, other needs were perceived as more pressing than a parenting focus. It may also explain the differing emphasis on parenting experienced by participants.

While some participants wanted more sessions, overall participants described significant improvements in well-being, suggesting ten sessions were sufficient. However, future studies could consider the addition of a follow-up session to provide a sense of closure and reassurance that patients are able to maintain changes independently.

### 4.3 Safety markers

The intervention was generally considered safe, as based on an overall improvement of participants' mental health, no participants moving from below to above clinical cut-off post-intervention, improved self-rated health, and minimal reported adverse effects, indicating no serious psychiatric morbidity as an adverse effect following the intervention. In previous studies, AFPT has also shown only minimal adverse effects (Lindqvist et al., 2020). Although not noted in the post-assessment scores, one participant described her well-being as worse after the intervention, and was encouraged to seek regular healthcare if necessary. Taken together, in line with previous research (Leichsenring et al., 2023), the benefits of AFPT appears to outweigh the harm of emotional distress during therapy.

### 4.4 Preliminary effects of the intervention

Symptoms of depression and anxiety were reduced after completion of the intervention and at follow-up, with a large proportion moving from above clinical cut-off to below pre-to-post intervention, indicating that participants mental health had tended to improve both in the short- and longer-term. This is in line with previous studies where guided self-help AFPT has been shown to be effective in decreasing symptoms of depression and anxiety in psychiatric populations (Johansson et al., 2017; Lindqvist et al., 2020). The effects of guided self-help AFPT on depressive symptoms and anxiety have also been found to be maintained or further improved at two-year follow-up (Johansson et al., 2017). In addition, parenting concerns decreased, while self-efficacy increased post-intervention, in line with previous research highlighting that these areas are important to address (Spiropoulos et al., 2023; Tavares et al., 2022; Matuszczak-Świgoń and Bakiera, 2021; Romare Strandh et al., 2025; Cessna et al., 2016; Moore et al., 2015). Closeness in the family showed a trend toward improvement, suggesting a potential impact that warrants further investigation in larger samples. These findings support the idea that parents' well-being positively influences other family members (Shin et al., 2023; Whisenant et al., 2023). One core aspect of AFPT is its focus on emotions. In this study, adaptive affective functioning appeared to improve from pre- to post-intervention, as measured by the affect-phobia test. Compared with a previous study (Lindqvist et al., 2020), which found significant gains in overall emotion regulation, the present study showed a tendency toward improvement in cognitive reappraisal, but a non-significant decrease in expressive suppression. These mixed results may reflect differences in measurement tools or variations in study procedures and populations. Nevertheless, the results indicate that AFPT may be preliminarily effective in improving a range of psychological measures in mothers with cancer.

### 4.5 Strengths and limitations

This study had multiple strengths. The study design included both quantitative pre-and post-assessments and qualitative interviews that enabled a comprehensive evaluation of the feasibility, acceptability and safety of the study procedures and intervention. Additionally, the use of validated instruments strengthens the reliability of the quantitative findings, while trustworthiness in the qualitative analysis was ensured by involving multiple authors throughout the process. Several factors in the present study increased generalisability and transferability of the results: the inclusion of participants independently of their geographical location and cancer diagnoses, both during and after completing cancer treatment. However, the study also had limitations, one being that the sample was considered relatively homogeneous, exclusively consisting of mothers, highlighting a commonly observed gendered pattern in engagement with psychotherapy trials (Nam et al., 2010). Parenting concerns in mothers may also differ from fathers who might experience needs for support differently (Matuszczak-Świgoń and Bakiera, 2021). While therapists were specialised and experienced in AFPT as a therapy method, the lack of a fidelity assessment of AFPT can also be considered a limitation of the study.

## 5 Conclusion

The results of this study suggest that AFPT via videoconferencing is feasible and acceptable for mothers diagnosed with cancer, and that it may result in overall improvements in mental health with minimal adverse effects. While these findings are promising, to confirm efficacy and explore long-term effects, a randomized controlled trial with longer-term follow-up, including a larger and more heterogeneous sample should be conducted.

## Author contributions

Conceptualization: LL, AW; Design: LL, AW; Acquisition: AK, MRS, GM, LL, AW; Analysis: AK, MRS, LL, AW; Interpretation of data: AK, MRS, GM, HL, YH, CS, LL, AW; Writing draft: AK, MRS; Reviewing draft: AK, MRS, GM, HL, YH, CS, LL, AW; Final approval: AK, MRS, GM, HL, YH, CS, LL, AW; Agreement to be accountable for all aspects of the work: AK, MRS, GM, HL, YH, CS, LL, AW.

## Funding

This work was founded by The Swedish Breast Cancer Association (grant number: F2024-0023). The funders had no role in study design, the collection, analysis and interpretation of data, in the writing of the report, or in the decision to submit the article for publication.

## Declaration of competing interest

The authors have no competing interests to disclose.

## Appendix A Supplementary data

Supplementary material Image 1
