# Supplementary material for: Affect-focused psychodynamic psychotherapy for mothers diagnosed with cancer – A feasibility study
Source: Internet Interv. 2026 Feb 16;43:100916. doi: 10.1016/j.invent.2026.100916 (PMC12934217; doi:10.1016/j.invent.2026.100916)
Supplement: Supplementary file 1 — Supplementary material [file mmc1.docx]

**Supplementary material**

**Semi-Structured Interview Guide**

***Inclusion***

*Example questions (followed by probing questions such as “Can you tell me more?” or “Can you give an example?”):*

- How did you receive information about the study?
- Was the information about the study sufficient?
- Is there anything that could be improved regarding the invitation to participate in the study?

***Experiences of the intervention***

*Example questions (followed by probing questions such as “Can you tell me more?” or “Can you give an example?”):*

- How did you experience participating in the treatment?
- Can you describe something positive about the treatment?
- Can you describe something negative about the treatment?
- How did you feel about the number of sessions and their frequency?
- What was it like to participate in the treatment online?
- How was the timing of the treatment in relation to your cancer diagnosis?
- Was there anything you felt was missing?

***Experience of Treatment Effects on Well-being***

*Example questions (followed by probing questions such as “Can you tell me more?” or “Can you give an example?”):*

- What were your expectations for participating in the study?
- Was the treatment relevant or not for your issues? If yes, in what way?
- Has the treatment affected your well-being positively or negatively?
- Have there been any effects or not on people around you, such as your child/children?
- Have you experienced any negative effects?

***Experience of the questionnaires and interview***

*Example questions (followed by probing questions such as “Can you tell me more?” or “Can you give an example?”):*

- How did you find answering the questionnaires?
- How much time did it take?
- Did you feel anything was missing?
- How was it to be interviewed about your participation in the study?

***Final Questions***

- How helpful was the intervention in meeting your needs, on a scale from 0-10 where 0 is not helpful at all and 10 is very helpful)
- Would you recommend this treatment to others in your situation? Why or why not?
- Is there anything that could be done to improve the intervention or the study procedures?
- Is there anything else you would like to share regarding your participation in the study?
